# Swedish Fertility Developments Before, During and After the COVID-19 Pandemic

**DOI:** 10.1007/s10680-025-09744-8

**Published:** 2025-07-21

**Authors:** Sofi Ohlsson-Wijk, Gunnar Andersson

**Affiliations:** Stockholm University Demography Unit (SUDA), Sociologiska Institutionen, Demografiska Avdelningen, Stockholm University, 106 91 Stockholm, Sweden

**Keywords:** Fertility, Fertility trends, COVID-19, Pandemic, Sweden

## Abstract

Many affluent societies saw a temporary increase in their fertility rates in 2021, during the COVID-19 pandemic. This included a number of countries that had experienced fertility decline during the 2010s, like the Nordic. In the immediate aftermath of the pandemic (2022–2023), fertility rates resumed their previous downward trend. Most research on the pandemic-related fertility trends has relied on aggregate data. Although a few studies have examined group-specific trends, hardly any have covered the post-pandemic years—an important step for revealing whether any uptick in 2021 had a lasting impact on fertility structures. Our study attends to this objective, with a focus on parity and group-specific fertility trends in Sweden before, during and after the COVID-19 pandemic. We apply event-history techniques to Swedish register data to unveil annual trends of birth risks in 2010–2022, for all Swedish-born women of childbearing age. First- and second-birth risks in 2015–2022 are analysed further across socio-demographic factors. Our study reveals that the “pandemic pattern” of fertility increase in 2021 and drop in 2022 was visible among subgroups with better possibilities to prepone already intended births. For example, the fertility increase and subsequent drop was particularly evident for mothers with young children and women with higher education and incomes. The pandemic fertility pattern reflects temporary changes in the timing of childbearing, more specifically a preponement of births that occurred in 2021 with resulting shortfall in 2022. The continued fall in fertility rates in 2023 should be viewed in the light of the long-term fertility decline.

## Introduction and Background

At the onset of the COVID-19 pandemic in 2020, Sweden and its Nordic neighbours had experienced more than a decade-long period of fertility decline (Comolli et al., 2021; Hellstrand et al., 2021; Ohlsson-Wijk & Andersson, 2022). This unanticipated development was shared by many other affluent societies, including other countries with previously relatively high fertility levels, such as France, the USA and the UK.

The COVID-19 pandemic brought an entirely new factor into this scenery, triggering a wide range of behavioural change including family behaviour. Many countries imposed lockdowns and school closures, which affected the everyday lives of parents, children and prospective parents. In Sweden, these restrictions were less drastic than in other countries. Pre-schools (for children aged 1–6) and primary schools stayed open, and people could still travel but were strongly advised to work from home whenever possible. Already at an early stage of the pandemic, researchers attended to the issue of the potential impact on childbearing behaviour and birth outcomes. Aassve & colleagues (2020, 2021) projected that socio-economic subgroups could be affected in different ways and that economic restraints in relation to the pandemic and increased feelings of uncertainty could lead to depressed fertility levels in affluent societies. Guetto et al. (2022) argued that beyond the role of uncertainty in driving down fertility levels in developed countries in recent decades, the pandemic was an additional layer of uncertainty that may depress fertility further.

Several comparative studies have continued to follow the development of fertility levels during the course of the pandemic and its immediate aftermath. Sobotka et al. (2023) present monthly data on total fertility rates for 38 high-income countries. They show that while the fertility rates in many countries temporarily declined nine months after the immediate onset of the pandemic, fertility rates later recovered and rather stayed at somewhat higher than pre-pandemic levels during much of 2021. The fertility rates in the Nordic countries appeared as some of the most resilient to any possible negative impacts of the pandemic on childbearing outcomes. Another turn in fertility developments, in the opposite direction, happened in early 2022 with fertility levels declining in many countries approximately nine months after spring 2021 when pandemic restrictions began to be lifted and vaccination programmes were rolled out on a broader basis. Jasilioniene et al. (2025) corroborate the findings on fertility declines in the aftermath of the pandemic but conclude that trend changes were far from universal. In many countries, they led to the return of fertility to their pre-pandemic levels. Bujard and Andersson (2024) focus on the Swedish case—and Germany—and demonstrate how aggregate fertility decline in these two countries indeed can be linked to the onset of the vaccination campaigns and the end of the most severe pandemic situation. They interpreted this trend change as the results of the vaccination programmes signalling a return to pre-pandemic living conditions, and perhaps the considerations of some women to postpone becoming pregnant until the time after a vaccination had been secured. Most previous research on actual fertility around the pandemic have relied on aggregate data and in some cases a set of macro-level indicators that could plausibly be related to pandemic-related fertility change (see also Nitsche et al. 2022; Winkler-Dworak et al., 2024). This is valuable when the focus is on cross-country comparisons and the broader perspective, but provides limited insight into the micro-level dynamics that have produced the aggregate fertility indicators that are studied. Nitsche and Wilde (2024) note that there is a need for more in-depth data to get at the many processes that contributed to fertility change during the pandemic and its immediate aftermath.

One example of a micro-level subgroup analysis is a study by Lappegård et al. (2024) with analyses of life-course data for Norway during the first year of the pandemic. They demonstrate a pattern of slightly elevated fertility in Norway after the onset of the pandemic that was mainly driven by the fertility change of women in their prime childbearing ages, women who were already mothers and the more highly educated as well as those employed in public administration. Similarly, Arnalds et al. (2025) found that in Iceland, third births ratios showed a clear uptick in 2021, and it was especially strong for those with high education and family income. These Nordic studies attribute the upward fertility change to people in more socially and economically secure circumstances that perceived the pandemic situation as an opportunity to prepone births that in many cases were already planned. Bujard and Andersson (2024) refer to the possibility that the more home-centred life situations during the pandemic could have contributed to slightly more childbearing as a “cocooning effect” in fertility behaviour, which has been corroborated by focus-group discussions in Iceland (Arnalds et al., 2025). In Finland, the 2021 upturn seemed more universally spread, as it was clearly visible for all parities and all ages apart from those below their mid-20s, as measured by aggregate rates (Nisén et al., 2022).

To gain further insight into patterns of pandemic and post-pandemic fertility change in an affluent society, we rely on individual life-course data from Swedish administrative registers. Our aim is to show how fertility levels developed on an annual basis before, during and in the immediate aftermath of the pandemic and whether patterns in behaviour have varied across a wide range of socio-demographic indicators.

## Swedish Fertility Developments in Context

**Fig. 1:**
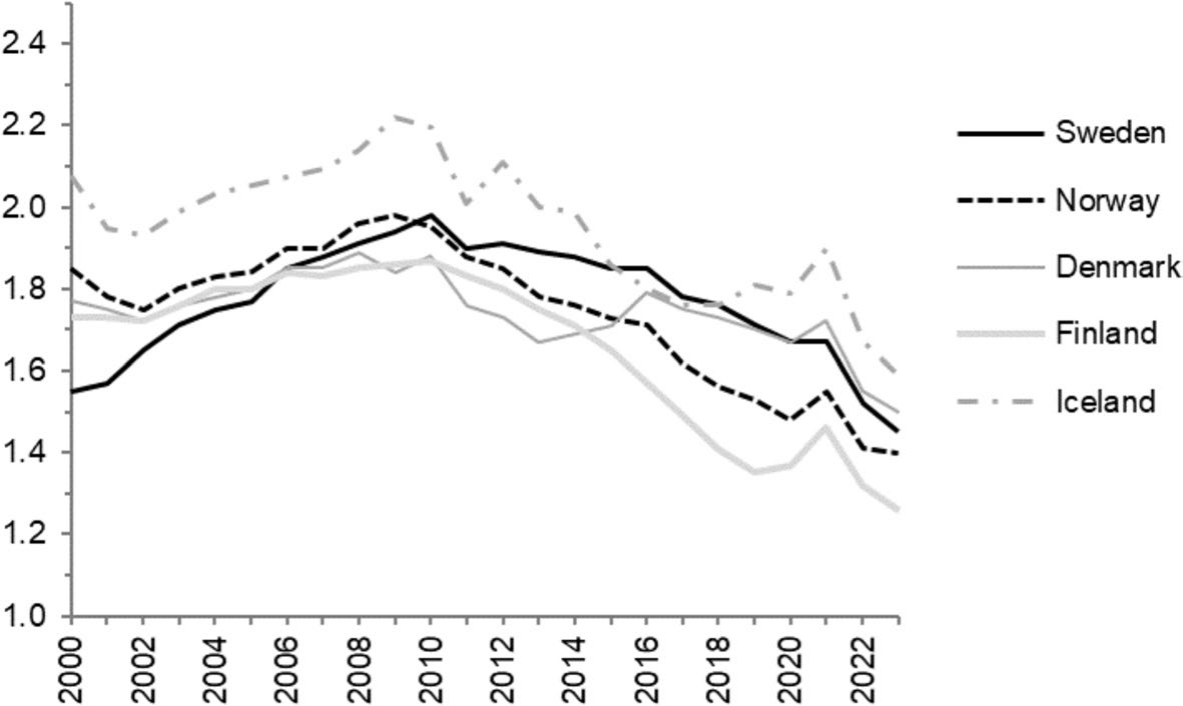
Total fertility rates of the nordic countries 2000–2023. *Source* Nordic Statistical Central Bureaus

Sweden experienced a temporary break in its long-term fertility decline during the pandemic year 2021 and a return to declining fertility levels in 2022, with aggregate fertility trends similar to those of its Nordic neighbours, as presented in Fig. 1. The Swedish pandemic trend changes were less pronounced than those in its Nordic neighbours and a number of other European countries, such as the German- and Dutch-speaking, but resembled those observed in many other European countries (see Fig. 7 in the Appendix). Not all European countries exhibited signs of pandemic influences on their TFR. The Swedish trend changes in fertility largely occurred at the shifts of the calendar years 2020–2021 and 2021–2022 (Bujard & Andersson, 2024), which makes a focus on annual changes in childbearing behaviour suitable for our purposes.

The unexpected, and largely still unexplained, fertility decline in Sweden and its Nordic neighbours that started around 2010 (see Fig. 1) continued in 2022 and 2023 after the temporary halt or reversal of the fertility declines in 2021. A previous study (Ohlsson-Wijk & Andersson, 2022) with data that stretched into 2018 shows that the Swedish fertility decline during the 2010s was confined to decreasing levels of first-birth rates but that the decline in these rates was otherwise strikingly similar across different socio-demographic groups of women and men. The first-birth declines were not limited to younger women, which indicates an increase in the propensity to remain childless rather than a mere postponement of entries into parenthood (Hellstrand et al., 2021).

A study by Cantalini et al. (2024) focuses on the formation of cohabiting and marital unions in Sweden. That study stretches into the pandemic period and shows that also marriage rates declined during the 2010s and dropped further during the pandemic. In contrast, the rates of cohabitation formation among young adults remained largely stable, also during the pandemic years. It thus shows that even with the different restrictions on people’s lives during the pandemic, young women and men continued to form couples and enter co-residential unions as before.

With access to life-course data on Swedish-born women, we aim to further disentangle the Swedish fertility trends around the time of the pandemic, by parity, age group and socio-demographic characteristics such as region of residence, parental migration background, educational attainment and labour-market status. With the increase in work from home during 2021, we expect that some socio-economically privileged groups may have been better positioned than others to take advantage of a more home-centred life situation as a suitable context to have a (next) child. We expect that this pattern could be more visible in the preponement of already planned births, such as the conceptions of an already anticipated second child. As people with a migration background were more negatively affected by the health and mortality outcomes of the pandemic (Kolk et al., 2022; Rostila et al., 2021), we may expect that their fertility considerations differed from those with a full Swedish background.

In short, our study is descriptive and aims at disentangling any differences in fertility outcomes and changes in behaviour during the course of the pandemic. We seek to establish (1) the extent to which fertility developments in 2021 differed between socio-demographic groups in the Swedish population, i.e. the extent to which there were any segmented responses to the pandemic in this population; and (2) whether any trend changes in 2021 were mainly of a temporary nature so that fertility patterns returned to their pre-pandemic structures in 2022.

## Data and Methods

### Data and Study Population

Our study covers trends in parity-specific fertility during 2010–2022. Further analyses of subgroups cover the period 2015–2022 and are confined to first and second births as these constitute the vast majority of birth outcomes, and we aim to avoid overloading the presentation of empirical results (data on higher parities available from authors on request). Empirically, our study partly extends the findings presented by Ohlsson-Wijk and Andersson (2022), which covered years up to 2018 with a focus on the decline in first births. Our analyses are based on Swedish register data covering the total resident population. The data are longitudinal and at the individual level, comprising family-demographic histories as well as additional socio-economic and geographic information. We focus on birth trends for women, as the childbearing patterns across various subgroups have been strikingly similar for women and men in recent years (Ohlsson-Wijk & Andersson, 2022). Only Swedish-born women are included, because the fertility of (newly arrived) immigrants depends largely on short-term influences related to their duration of residence in the country (Andersson, 2004; Mussino et al., 2021). This may create quite unstable period estimates of fertility for the migrant population, which are shaped by factors relating to the migration process (age at arrival, duration of residence, country of emigration, reason for migration) that merit separate investigation. However, we measure the role of migration background through information on the parents of women in the second generation in Sweden.

### Methods

The register data are analysed using event-history techniques, more specifically piecewise constant baseline intensity models. In each model, the variable of interest is interacted with calendar year to display period fertility trends across the different variable categories. Birth risks are calculated with monthly precision. The clock for first births starts in the month a woman turns age 16, and the clock for higher-order births starts at the month of the last previous birth. Women are censored at whichever occurs first of the month of first emigration, turning age 46, death, or the end of December 2022. Higher-order births are also censored 10 years after the birth of the last previous child.

### Variables

‘Calendar year’ is measured in single full years during 2010–2022, and ‘age’ refers to the age at the end of each calendar year. The ‘region of residence’ is measured at the end of the previous year, where the 290 municipalities of Sweden are divided into (1) large cities (Stockholm, Gothenburg, Malmö), (2) commuting municipalities near these large cities, (3) medium-sized cities, (4) small towns, (5) municipalities near medium-sized cities or small towns, and (6) rural municipalities (Swedish Association of Local Authorities & Regions, 2021). ‘Migration background’ covers the birth country of the woman’s parents, with four categories based on degree of proximity to Sweden: (1) both parents Swedish-born, (2) both parents Nordic-born (non-Swedish) or one Nordic and one Swedish, (3) both parents European-born (non-Nordic) or one European and one Swedish/Nordic, and (4) both parents born outside Europe or one born outside Europe and one Swedish/Nordic/European.

‘Education’ is measured in accordance with ISCED-97 as primary, secondary, or tertiary level in the previous year. In the same manner, ‘labour-market activity’ measures the main activity in the previous year. It has eight categories based on work-related earnings before tax and unemployment or student activity. Those classified as mainly working also include women on paid parental leave or temporary sick leave. They are divided into five categories, with lower-income bounds established in the earnings profiles measured in 2020^1^ as follows: low earnings (€9021), medium-low (€20,664), medium (€28,455), medium-high (€34,663), or high earnings (€43,264). The bounds for the income categories are adjusted for inflation and therefore vary across years, enabling comparison over time. The other three categories are students, unemployed, and non-participants. The students have study allowances as their main source of income, whereas the unemployed have unemployment benefits as their main source, and both groups receive less than the threshold for medium-low income in work-related earnings. (If earning more, they are categorised as mainly in paid work.) Women are categorised as non-participants if they receive neither student allowances nor unemployment benefits and their work-related earnings are zero or less than “low income”. The analyses of higher-order births also include the variables ‘birth order’ and ‘time since the previous birth’.

## Findings

**Fig. 2:**
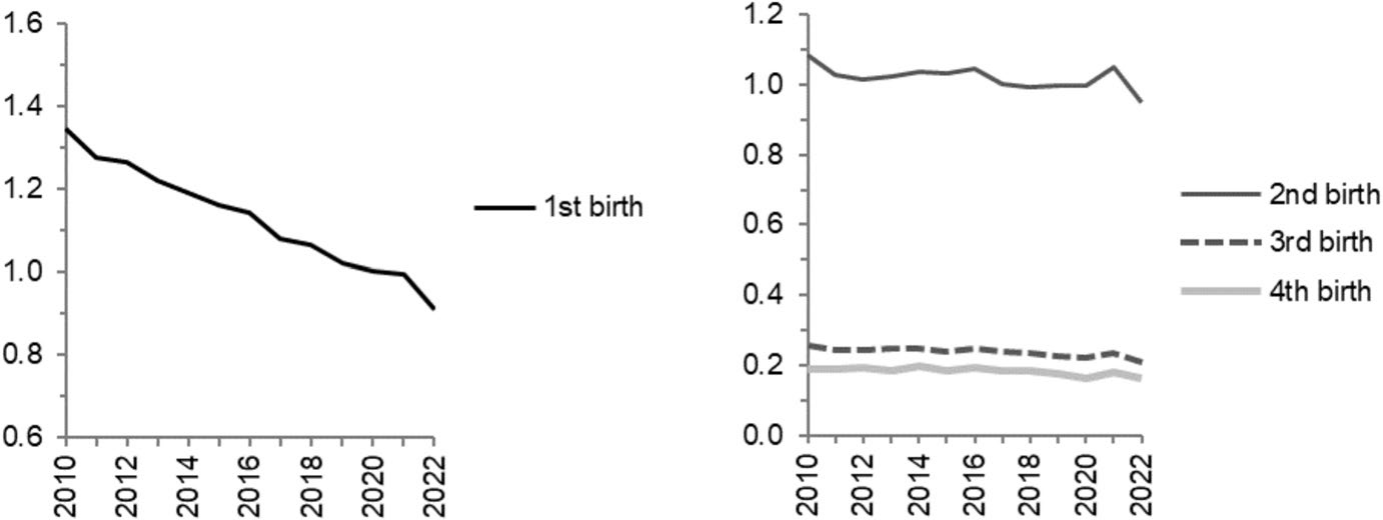
Relative birth risks by parity across calendar years 2010–2022. Separate models for first and higher-order births. For parities 2–4, birth order is interacted with calendar year. Swedish-born women. First-birth risks are standardised by age, with the year 2020 as reference category. Higher-order births are standardised by age group and time since last birth, with reference category second births in 2020

The calendar year indices by parity and socio-demographic characteristics are presented below, beginning with the trends in parity-specific fertility in Fig. 2. The decline in first births and stability for higher parities that have previously been documented for years up to 2018 (Ohlsson-Wijk & Andersson, 2022), are also evident in the four subsequent years, but of most interest for this study are the visible deviations from these underlying trends during the pandemic. Overall, in many of our findings there is an unexpectedly high level in 2021 and a decline to a visibly lower level in 2022, compared with the overall trend in the preceding years (2015–2020). Hereafter, we refer to this as the “pandemic pattern”.

In 2021, there was a halt in the decline for first-birth risks with a subsequent sharper fall in 2022. For higher-order births, there was a bump in birth risks in 2021 followed by a reduced level in 2022, although this is not as easily observable for third and fourth births in this figure (see also Fig. 10b in the Appendix) as for second births, as those parity transitions occur at much lower levels than second births. Figure 10 in the Appendix also exhibits fertility trends over calendar years for the main covariates analysed in this section, but with the year 2020 being used as reference year for each variable category. The purpose of this extension is to better highlight the relative changes for each subgroup during the pandemic period.

**Fig. 3:**
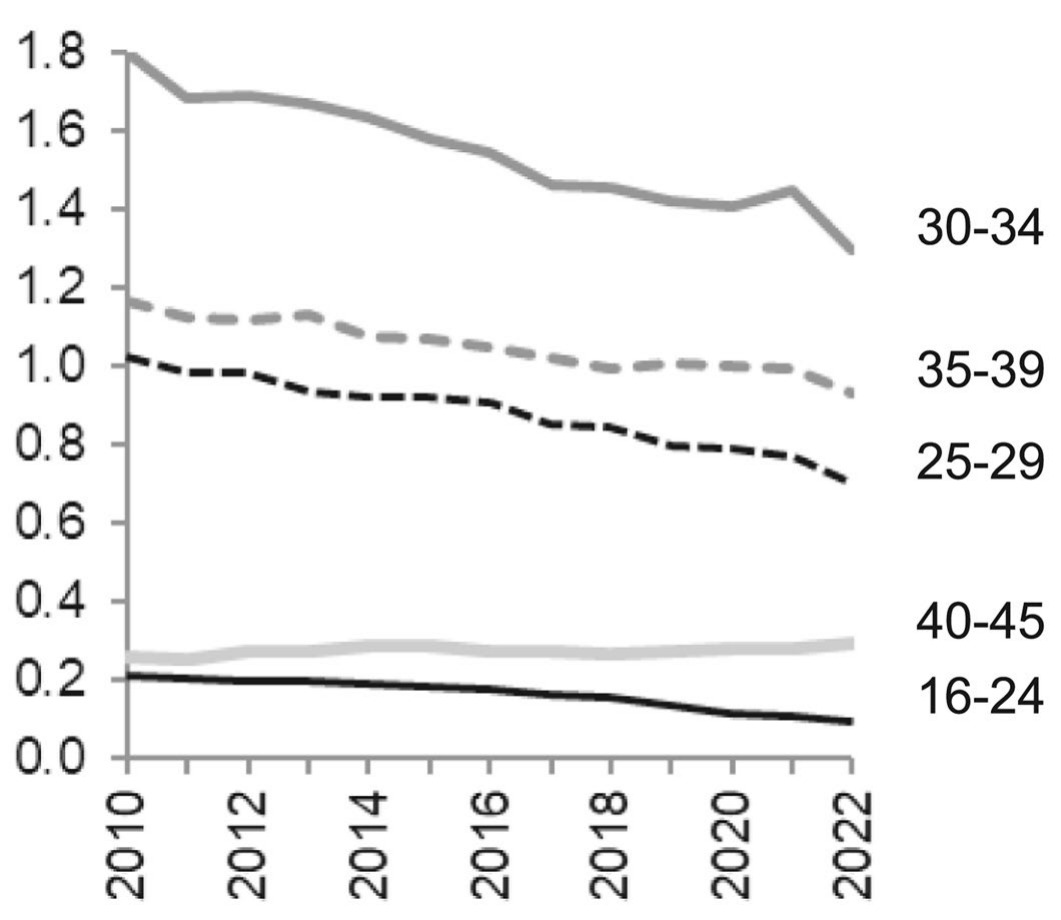
Relative first-birth risks by age groups across calendar years 2010–2022. Interaction of age group and calendar year. Swedish-born women. Reference category is ages 35–39 in the year 2020

The following figures display the trends in childbearing across subgroups for first and second births, which are the predominant parity transitions. In Fig. 3, age-specific first-birth risks are presented, showing that the halt in the decline of first births in 2021 was primarily driven by behavioural changes among women in their peak childbearing ages (30–34), who had a slight increase from 2020 to 2021 and a fall-back in 2022. Other age groups seem unaffected by the pandemic.

**Fig. 4:**
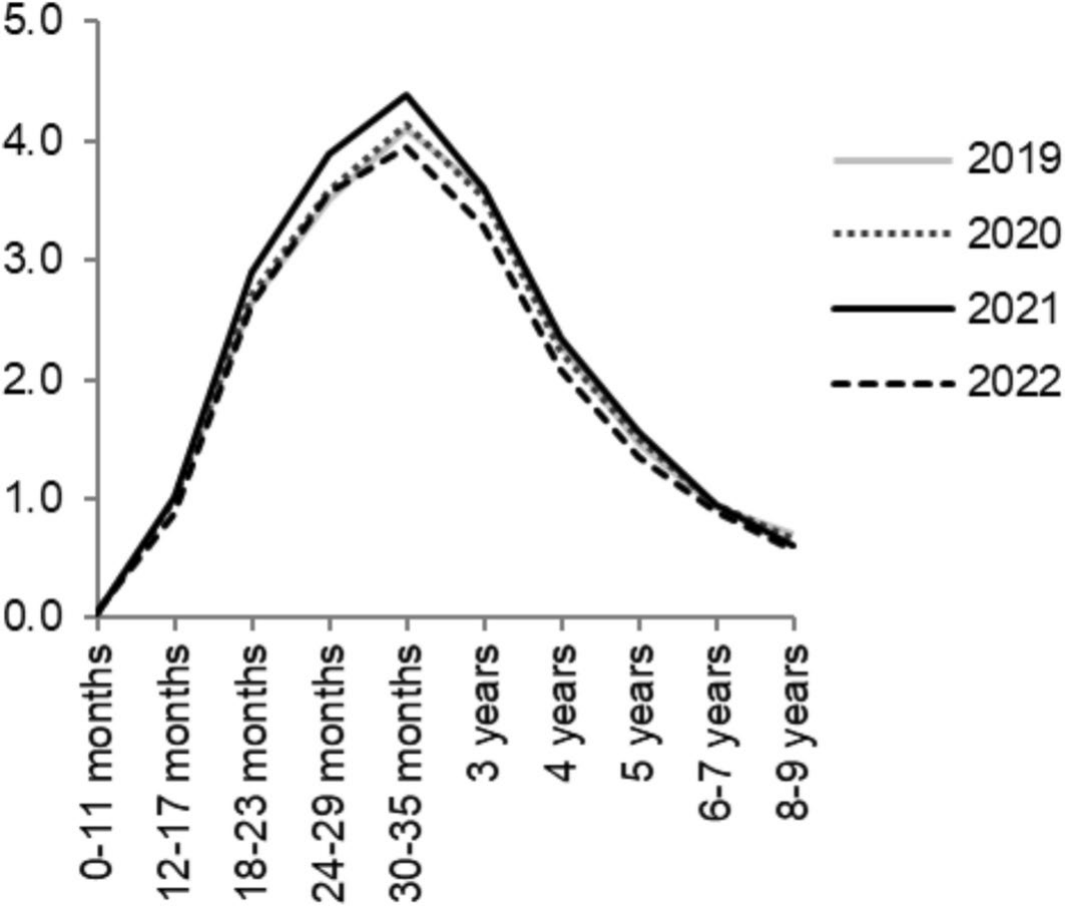
Relative second-birth risks by time since last birth and calendar years. Interaction of time since last birth and calendar year. Swedish-born women. Reference category is 12–17 months in the year 2020. Standardised by age of woman. In order to increase readability, we do not include the years 2015–2018, but they are nearly identical to 2019 and 2020

Figure 4 displays second-birth risks by time since the previous birth and calendar year. In this figure only, the x-axis is time since last birth, whereas calendar year is represented by the different lines. It indicates that the elevated second-birth risks in 2021 were primarily driven by an increase in childbearing at relatively short birth intervals, when the first child was between 1.5 and less than three years old. The reduction of second-birth risks in 2022 was primarily driven by lower birth risks at slightly longer durations. Therefore, it seems that the overall increase in 2021 and the drop in 2022 were largely the result of the preponement of second births in 2021. For example, instead of waiting three years to have the second child (resulting in a birth in 2022), many couples had their second child after only two years (with a birth in 2021).

**Fig. 5:**
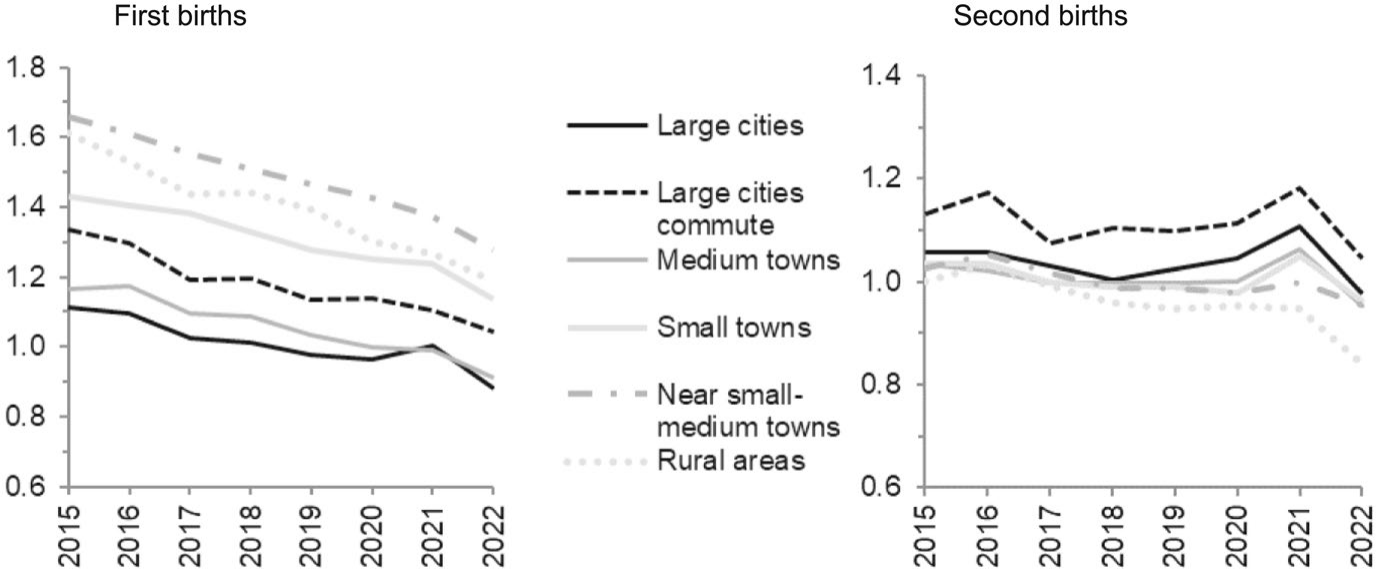
Relative first- and second-birth risks by regions across calendar years. Interaction between region and calendar year. Swedish-born women. First and second births modelled separately. Reference category medium towns in 2020. First births standardised by age of woman, and second births standardised by age of woman and time since last birth

The patterns across types of regions are presented in Fig. 5 and reveal that for first births, it was primarily women in the three largest cities that changed their childbearing behaviour during the pandemic, with higher intensities in 2021 followed by a fall-back in 2022. (The lower absolute levels of first-birth risks in this category are largely due to the higher average age of becoming a parent in the largest cities.) For second births, the behavioural change was more spread across the country, with a clear bump in 2021 and fall-back in 2022. It was only the rural areas and municipalities near small- or medium-sized towns that showed no evident response to the pandemic.

The findings for parental migration background (see Appendix Figs. 8 and 10e) show a pandemic fertility pattern for women of all backgrounds and for first and second births alike, with the exception of first births for women with Nordic-born parents. The levels of second-birth risks are higher for women with a full Swedish background than for those with a migration background through their parents, but the increase in second-birth risks in 2021 and the fall-back in 2022 were observed across all groups of one-child mothers.

**Fig. 6:**
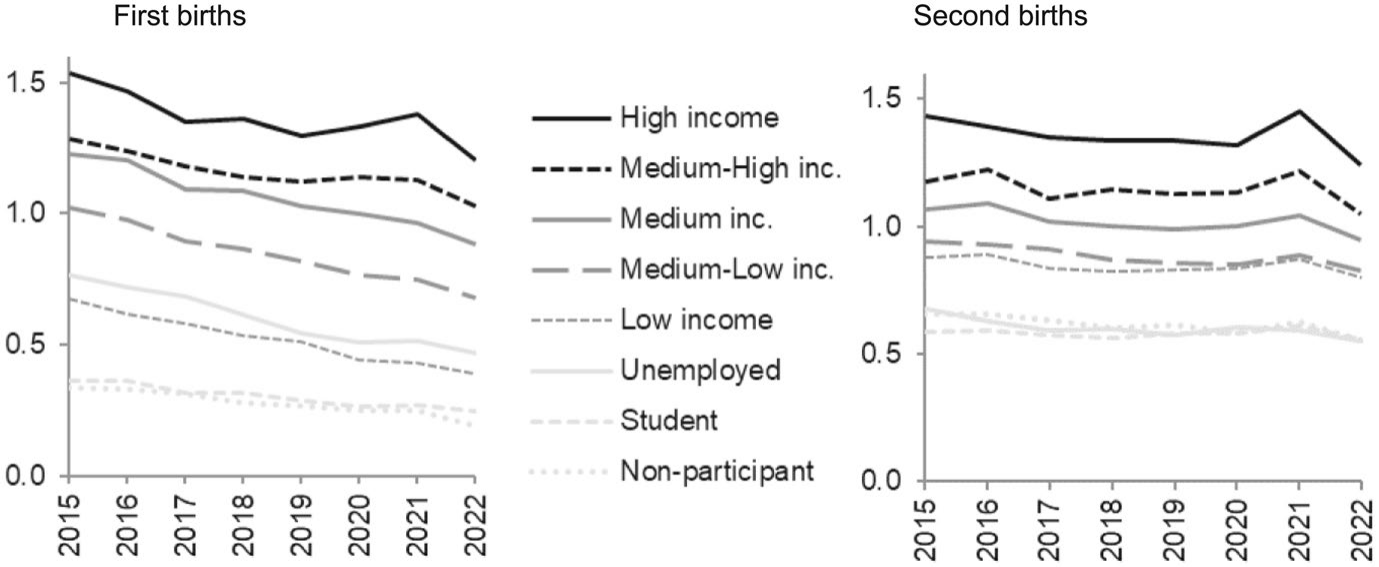
Relative first- and second-birth risks by labour-market activity across calendar years. Interaction between labour-market activity and calendar year. Swedish-born women. First and second births modelled separately. Reference category medium income in 2020. First births standardised by age of woman, and second births standardised by age of woman and time since last birth

**Fig. 7:**
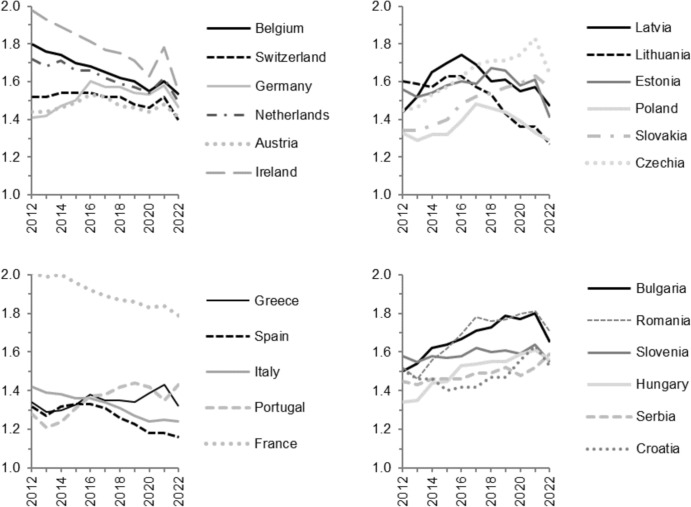
Total Fertility Rates of selected European countries 2012–2022. Countries are included in the figure if they are represented in the Eurostat TFR data for all years 2012–2022, and have at least 1 million inhabitants. Potential break in time series in any of the years 2019–2022 reported for Czechia 2020, Hungary 2022, Italy 2019, Poland 2022 and Serbia 2022 *Source* Eurostat, 2025

**Fig. 10:**
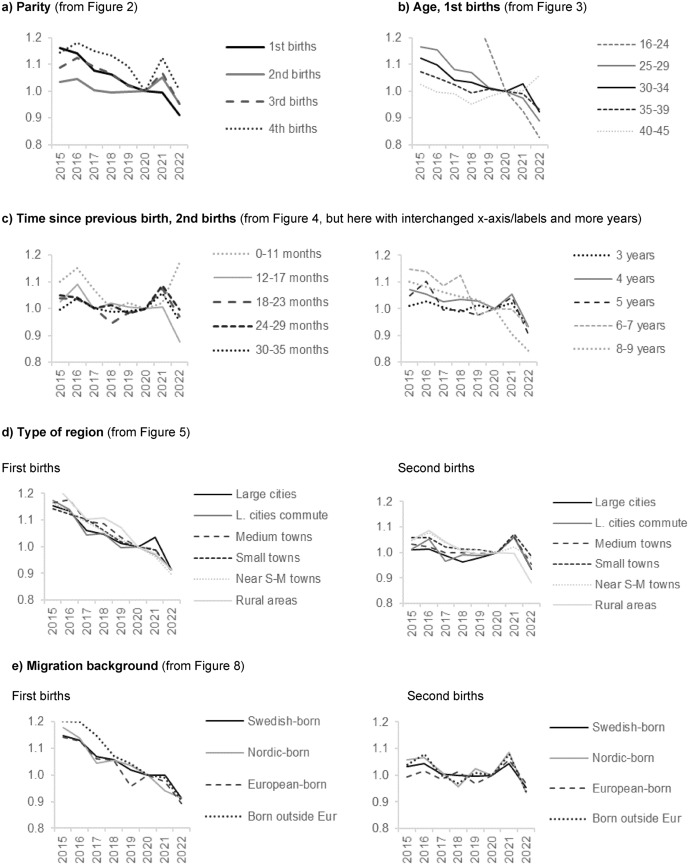

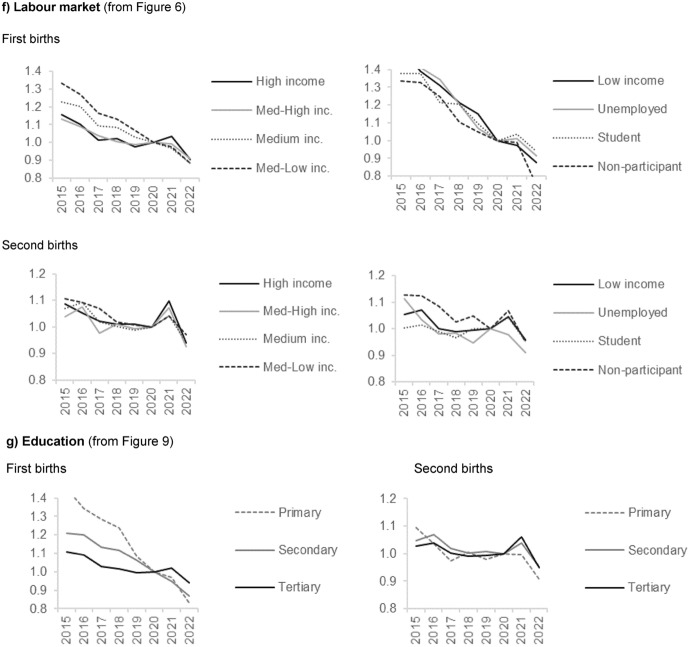
Relative birth risks across subgroups, with 2020 as reference category for each subgroup, based on models presented above

Fertility trends by women’s type of labour-market activity are presented in Fig. 6. It shows that the elevated levels of first-birth risks in 2021 were mainly attributable to the changing behaviour among those with the highest incomes. For second births, again, the pandemic pattern was more spread across groups and visible for all groups of women who were established with earnings in the labour market, although strongest among the high-income earners. Further inspection of relative levels for each labour-market category (Appendix Figure 10f) shows a temporary peak in 2021 also for some of the groups outside the labour market—for first births: students and unemployed; for second births: students and non-participants. Evidently, some among the least advantaged strata, despite overall very low fertility, also perceived this as a more suitable time to have children.

Analyses of first- and second-birth risks by women’s educational attainment (Appendix Figs. 9 and 10g) support the findings by labour-market activity. The bump in first-birth risks in 2021 and subsequent decline were mainly observed among tertiary educated women, whereas for second births, the pattern was also visible among those with secondary education.

Region of residence, educational level, and labour-market activity are often interrelated, as for example high-income earners usually are more highly educated and more often live in the largest cities. Many of them are office workers with more flexible work arrangements and larger possibilities to work from home during the pandemic. To account for this, we conducted additional analyses (available upon request) where—for each factor region of residence, educational attainment and labour-market activity)—the other two were included as controls. In some cases, this changed the relative levels between categories, but the pandemic patterns were nearly identical to those presented here. Thus, the regional and socio-economic patterns across the pandemic held independently of each other. Furthermore, partnering is positively related to socio-economic status for Swedish women and men (Cantalini et al., 2024). Therefore, we added a variable indicating whether women co-resided with a partner (married or cohabiting) to the sensitivity analysis of first-birth risks where region, education and labour-market activity were included (available upon request). This did not change the overall conclusions about pandemic patterns across subgroups, which means that changes in partnership status were not driving the pandemic patterns across socio-economic groups.

## Discussion

In our study, we focused on the case of Sweden to examine the structure of fertility change before, during and shortly after the COVID-19 pandemic of 2020–21. We relied on analyses of longitudinal life-course data from Swedish administrative registers to unravel the structures in childbearing behaviour that produced slightly elevated aggregate fertility levels during the pandemic year 2021 and a subsequent fall-back in 2022. The pre-pandemic trends extend from those previously documented by Ohlsson-Wijk and Andersson (2022), while we observed a clear but non-dramatic deviation from these pre-pandemic trends for births that occurred around nine months after the peak of the pandemic in 2020 and early 2021, i.e. during 2021. We identified a clear “pandemic pattern” with unexpectedly high levels of birth risks for several groups of women during 2021 and a subsequent fall-back in 2022 as compared to the overall trends in the preceding period. We interpret our findings as the effects of temporary changes in the timing of childbearing during the pandemic, namely a preponement in 2021 with a resulting shortfall and return to previous patterns in 2022.

The pandemic pattern was visible in several population subgroups, but much more widespread for second-birth progressions than for becoming a parent. For first births, the pandemic pattern was confined to women in their early 30s, which are the prime childbearing ages when women are particularly likely to already have firm intentions to have a child in the near future. The modest pandemic elevation in first births was located to women living in the largest cities of Sweden, those who were highly educated and had the highest earnings. These groups may to a larger extent than others be situated in workplaces and occupations where it was possible or even commanded to work from home, leading to reduced commuting time and potentially more time spent with their partner. They probably also had material circumstances that facilitated the preponement of becoming a parent. Sensitivity analyses showed that these socio-economic pandemic patterns were not due to increased shares of partnered women among those with high education or income.

Curiously, there was also a temporary elevation of first- and second-birth risks for some groups outside the labour market. This could signal that they saw having children as a strategy for reducing uncertainty (see, for example, Friedman et al., 1994), during particularly uncertain circumstances arising from the combination of non-employment and the pandemic. Important to note here is that childbearing is overall rare among those who are not active on the labour market in Sweden, which indicates that this mechanism is not strong in general, and it also indicates that their patterns may be particularly sensitive to random variation.

For second births, we revealed a more pervasive pattern of speeding up the transition to a next child during the pandemic. In 2021, compared to preceding years, there were higher shares of women who had the second child when their first child was 1.5 to not yet three years old, instead of waiting a year or so longer. This subsequently led to lower shares of women having their second child when the first child was around 2.5 to four years old, in 2022. In Sweden, there is a strong two-child norm and the second child often follows the first within a relatively narrow time frame of a few years. There is arguably a lower threshold for preponing births among those who already have had one child than for those who would transition from being childless to becoming a first-time parent.

In line with this, the pandemic pattern of elevated fertility in 2021 was evident among a much wider range of subgroups for second births than for first births. This pattern could be observed for all types of regions in Sweden except for rural areas and municipalities outside small- or medium-sized towns. It also appeared for both tertiary and secondary educated women and for all income groups of women established in the labour market, although it was slightly stronger for those with the highest education and earnings. Thus, there seemed to be a fertility response to the pandemic among all groups of one-child mothers apart from those in the most sparsely populated areas where the pandemic may have had a different impact on everyday life. In countries that do not have as truly sparsely populated regions as Sweden, the pandemic effects on fertility (and everyday life) may perhaps be detected across all parts of the country. The pandemic effect on childbearing was observed irrespective of parental migration background, for both first and second births, except for childless women of non-Swedish Nordic descent.

Another potential explanation for the weaker fertility peak among women with lower labour income or education could be health-related issues, such as higher risks of COVID-19 infection among those in certain service or transport occupations with much social interaction (Folkhälsomyndigheten 2020). Other health-related concerns, such as fears of an overwhelmed healthcare system and considerations of foetal development, have been identified as potential deterrents to having children during the pandemic (Raybould et al., 2023). Nevertheless, although the pandemic was in essence a health crisis, it impacted everyday life in a wide range of ways, which may have affected childbearing patterns beyond any role of health concerns. Contrary to expectations, aggregate levels of conceptions were not negatively related to the COVID-19 incidence rates and mortality rates, for example in Sweden (Bujard & Andersson, 2024). Thus, overall fertility increased temporarily, not decreased, during the pandemic.

An overarching conclusion is that the subsequent fertility decrease in 2022 was primarily produced by groups that had experienced a relative increase in 2021. The decline in first-birth rates in 2022 was partly a continuation of the previous pre-pandemic trend of fertility decline. The decline in second-birth rates primarily reflected a return to the patterns in behaviour that held before the onset of the pandemic. All in all, our findings indicate a visible tempo effect of the pandemic on fertility levels and fertility timing, but no apparent lasting effect on fertility outcomes in its immediate aftermath. At the end of the pandemic, Sweden still experienced a situation with a decline in first-birth rates and relative stability in higher-order births—with no apparent lasting effect of the pandemic on childbearing behaviour in the short run. The decline in the total fertility rate continued in 2023, hitting a historical low and with the underlying structures yet to be uncovered.

Comparative research shows that the pandemic pattern of slightly increased childbearing during 2021 and the subsequent fall-back in 2022 was shared also by many other countries than Sweden (Jasilioniene et al., 2025; Sobotka et al., 2023), not least the Nordic neighbours. Subgroup analyses from the Nordic countries reveal developments similar to the Swedish. Nisén et al. (2022) has shown that the increase in TFR in 2021 for Finland was stronger in the capital region, which resonates well with the elevated first-birth risks we found for the large cities of Sweden. A study focusing on third births in Iceland (Arnalds et al., 2025) corroborates the positive role of education and income for the 2021 uptick there. It could be argued that working from home during the pandemic would impact work–life balance positively particularly in countries like Sweden and Iceland where pre-schools and primary schools stayed open, which was not the case in many other European countries. For example in Poland, working from home during the pandemic was generally related to lower fertility intentions among mothers (Kurowska et al., 2023). Nevertheless, also Norway, where (pre-) schools closed, had a clear pandemic fertility increase among the socio-economically advantaged groups. The socio-demographic groups that contributed to the slightly elevated fertility during 2021 in Norway were largely the same as those we identified for Sweden, in terms of age, parity and socio-economic position, although the post-pandemic period was not covered in that study (Lappegård et al., 2024). It is remarkable that the structure of fertility change was so similar in these countries given that the pandemic restrictions on everyday life were more severe in Norway than in Sweden (Lappegård et al., 2024). Whether the identified socio-demographic patterns are particular for the Nordic setting, with high public spending, family-friendly policies and high gender equality, or also hold for other countries, remains for future research to determine.

The main contribution of our study is that we demonstrated that the COVID-19 pandemic seems to have left such a non-lasting effect on Swedish fertility trends and childbearing behaviour in its immediate aftermath. Already at the time when people could see the end of the pandemic, including with the introduction of large-scale vaccination programmes (Bujard & Andersson, 2024), fertility trends and fertility patterns appeared to return to their pre-pandemic structures. A further observation is that the changes in fertility levels during the peak of the pandemic were rather modest in Sweden, even if the structure in the change that occurred was very clear. It is perhaps remarkable that an intervention with such a profound impact on people’s lives and living conditions as the pandemic and its related changes in living conditions appears to have left little impression on childbearing behaviour. The pandemic may still have left enduring imprints, within the broader context of increasing global uncertainties and general fertility decline.

## Figures and Tables

**Fig. 8:**
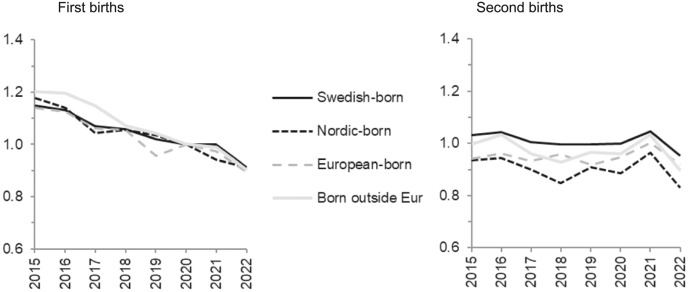
Relative first- and second-birth risks by (parental) migration background across calendar years. Interaction between migration background and calendar year. Swedish-born women. First and second births modelled separately. Reference category Swedish-born, 2020. First births standardised by age of woman, and second births standardised by age of woman and time since last birth

**Fig. 9:**
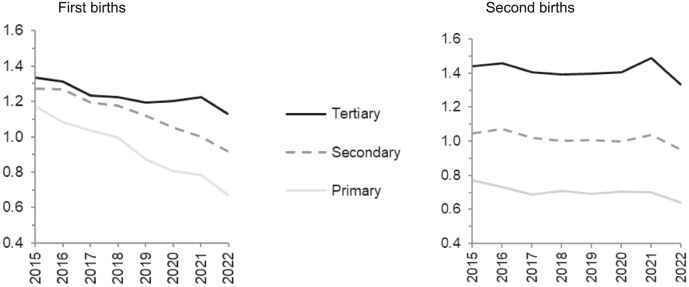
Relative first- and second-birth risks by education across calendar years. Interaction between education and calendar year. Swedish-born women. First and second births modelled separately. Reference category secondary education in 2020. First births standardised by age of woman and second births standardised by age of woman and time since last birth

## Data Availability

This study is based on de-identified full-population register data at the individual level, which may be regarded as sensitive and are therefore protected in full compliance with the General Data Protection Regulation (GDPR). The data are located at the servers of Statistics Sweden, which are accessed remotely through their secure Micro-Online Access System (MONA). Individual-level data may not be exported from that server. The user must be approved by Statistics Sweden by meeting certain criteria, such as being affiliated with an established institution and being a participant of a specific project approved by the ethics authority.

## Appendix

See Figs. 7, 8, 9 and 10.

## Footnotes

1 The bounds are based on the income distribution of all women aged 16–49 in 2020, irrespective of parity. For women earning more than a minimum threshold corresponding to a level of €9,021 annually, five equally large income categories are created (quintiles). Exchange rates to Euros are based on the annual average for 2020.

## References

[CR1] Aassve, A., Cavalli, N., Mencarini, L., Plach, S., & Livi Bacci, M. (2020). The COVID-19 pandemic and human fertility. *Science,**369*(6502), 370–371. 10.1126/science.abc9520 32703862 10.1126/science.abc9520

[CR2] Aassve, A., Cavalli, N., Mencarini, L., Plach, S., & Sanders, S. (2021). Early assessment of the relationship between the COVID-19 pandemic and births in high-income countries. *Proceedings of the National Academy of Sciences of the United States of America (PNAS)*. 10.1073/pnas.2105709118 10.1073/pnas.2105709118 PMC8433569 34462356

[CR3] Andersson, G. (2004). Childbearing after migration: Fertility patterns of foreign-born women in Sweden. *International Migration Review,**38*(2), 747–775. 10.1111/j.1747-7379.2004.tb00216.x

[CR4] Arnalds, Á., Jónsson, A. K., & Símonardóttir, S. (2025). The 2021 Baby boom in Iceland: Exploring the role of a parental leave reform and the COVID-19 pandemic. *European Journal of Population*. 10.1007/s10680-024-09727-1 10.1007/s10680-024-09727-1 PMC11729602 39804448

[CR5] Bujard, M., & Andersson, G. (2024). Fertility declines near the end of the COVID-19 pandemic: Evidence of the 2022 birth declines in Germany and Sweden. *European Journal of Population,**40*, 4. 10.1007/s10680-023-09689-w 38252183 10.1007/s10680-023-09689-w PMC10803721

[CR6] Cantalini, S., Ohlsson-Wijk, S., & Andersson, G. (2024). Cohabitation and marriage formation in times of fertility decline: The case of Sweden in the twenty-first century. *European Journal of Population,**40*, 15. 10.1007/s10680-024-09703-9 38777964 10.1007/s10680-024-09703-9 PMC11111655

[CR7] Comolli, C., Neyer, G., Andersson, G., Dommermuth, L., Fallesen, P., Jalovaara, M., Jónsson, A., Kolk, M., & Lappegård, T. (2021). Beyond the economic gaze: Childbearing during and after recessions in the Nordic countries. *European Journal of Population,**37*, 473–520. 10.1007/s10680-020-09570-0 33230356 10.1007/s10680-020-09570-0 PMC7676408

[CR8] Eurostat. (2025). Database (Fertility rates by age). https://ec.europa.eu/eurostat/databrowser. Retrieved 2025–01–31.

[CR9] Folkhälsomydigheten. (2020). Förekomst av COVID-19 i olika yrkesgrupper: Bekräftade COVID-19 fall i Sverige 13 mars – 27 maj 2020. [Prevalence of COVID-19 in different occupations: Confirmed COVID-19 cases in Sweden 13 March – 27 May 2020] Report No.: 20099.

[CR10] Friedman, D., Hechter, M., & Kanazawa, S. (1994). A theory of the value of children. *Demography,**31*(3), 375–401. 10.2307/2061749 7828763

[CR11] Guetto, R., Bazzani, G., & Vignoli, D. (2022). Narratives of the future and fertility decision-making in uncertain times. An application to the COVID-19 pandemic. *Vienna Yearbook of Population Research,**20*, 223–260.

[CR12] Hellstrand, J., Nisén, J., Miranda, V., Fallesen, P., Dommermuth, L., & Myrskylä, M. (2021). Not just later, but fewer: Novel trends in cohort fertility in the Nordic countries. *Demography,**58*(4), 1373–1399. 10.1215/00703370-9373618 34251453 10.1215/00703370-9373618

[CR13] Jasilioniene, A., Jasilionis, D., Jdanov, D., & Myrskylä, M. (2025). Association between the COVID-19 vaccination campaign and fertility trends: A population-level time series analysis for 22 countries. *BMJ Public Health,**3*, Article e001410. 10.1136/bmjph-2024-001410 40017921 10.1136/bmjph-2024-001410 PMC11842981

[CR14] Kolk, M., Drefahl, S., Wallace, M., & Andersson, G. (2022). Excess mortality and COVID-19 in Sweden in 2020: A demographic account. *Vienna Yearbook of Population Research,**20*, 317–348.

[CR15] Kurowska, A., Matysiak, A., & Osiewalska, B. (2023). Working from home during Covid-19 pandemic and changes to fertility intentions among parents. *European Journal of Population,**39*, 32. 10.1007/s10680-023-09678-z 37847441 10.1007/s10680-023-09678-z PMC10581933

[CR16] Lappegård, T., Kornstad, T., Dommermuth, L., & Kristensen, A. P. (2024). Understanding the positive effects of the COVID-19 pandemic on women’s fertility in Norway. *Population and Development Review*. 10.1111/padr.12539

[CR17] Mussino, E., Wilson, B., & Andersson, G. (2021). The fertility of immigrants from low fertility settings: Adaptation in the tempo and quantum of childbearing? *Demography,**58*(6), 2169–2191. 10.1215/00703370-9476273 34568893 10.1215/00703370-9476273

[CR18] Nisén, J., Jalovaara, M., Rotkirch, A., & Gissler, M. (2022). Fertility recovery despite the COVID-19 pandemic in Finland? *Finnish Journal of Social Research,**15*, 25–44. 10.51815/fjsr.120361

[CR19] Nitsche, N., & Wilde, J. (2024). Fertility and family dynamics in the aftermath of the COVID-19 pandemic. *Population and Development Review,**50*(S1), 9–22. 10.1111/padr.12648

[CR20] Nitsche, N., Jasilioniene, A., Nisén, J., Li, P., Kniffka, M. S., Schöley, J., Andersson, G., Bagavos, C., Berrington, A., Čipin, I., Clemente, S., Dommermuth, L., Fallesen, P., Galdauskaite, D., Jemna, D., Lerch, M., McDonnell, C., Muller, A., Neels, K., Pötzsch, O., Ramiro, D., Riederer, B., te Riele, S., Szabó, L., Toulemon, L., Vignoli, D., Zeman, K., and Žnidaršič, T. (2022). Pandemic babies? Fertility in the aftermath of the first Covid-19 wave across European regions. MPIDR Working Paper WP 2022–027. 10.4054/MPIDR-WP-2022-027

[CR21] Ohlsson-Wijk, S., & Andersson, G. (2022). Disentangling the Swedish fertility decline of the 2010s. *Demographic Research,**47*, 345–358. 10.4054/DemRes.2022.47.12

[CR22] Raybould, A., Mynarska, M., & Sear, R. (2023). “The future is unstable”: Exploring changing fertility intentions in the United Kingdom during the COVID-19 pandemic. *Perspectives on Sexual and Reproductive Health,**55*(4), 229–238. 10.1111/psrh.12248 38084828 10.1111/psrh.12248

[CR23] Rostila, M., Cederström, A., Wallace, M., Brandén, M., Malmberg, B., & Andersson, G. (2021). Disparities in Coronavirus disease 2019 mortality by country of birth in Stockholm, Sweden: A total-population-based cohort study. *American Journal of Epidemiology,**190*(8), 1510–1518. 10.1093/aje/kwab057 33710317 10.1093/aje/kwab057 PMC7989658

[CR24] Sobotka, T., Zeman, K., Jasilioniene, A., Winkler-Dworak, M., Brzozowska, Z., Alustiza-Galarza, A., Németh, L., & Jdanov, D. (2023). Pandemic roller-coaster? Birth trends in higher-income countries during the COVID-19 pandemic. *Population and Development Review*. 10.1111/padr.12544

[CR25] Swedish Association of Local Authorities and Regions. (2021). Classification of Swedish municipalities 2017 [electronic resource]. Stockholm: Swedish Association of Local Authorities and Regions. https://skr.se.

[CR26] Winkler-Dworak, M., Zeman, K., & Sobotka, T. (2024). Birth rate decline in the later phase of the COVID -19 pandemic: The role of policy interventions, vaccination programmes, and economic uncertainty. *Human Reproduction Open,**2024*(3), hoae052. 10.1093/hropen/hoae052 39345877 10.1093/hropen/hoae052 PMC11438547

